# Supplementary material for: A Critical View of the Application of the APEX Software (Aqueous Photochemistry of Environmentally-Occurring Xenobiotics) to Predict Photoreaction Kinetics in Surface Freshwaters
Source: Molecules. 2019 Dec 18;25(1):9. doi: 10.3390/molecules25010009 (PMC7017383; doi:10.3390/molecules25010009)
Supplement: Supplementary file 1 [file molecules-25-00009-s001.zip › 3.2.4_gcc-4.4.0.zip!/3.2.4_gcc-4.4.0/3.2.4_gcc-4.4.0/doc/octave/html/interpreter/Functions-of-a-Matrix.html]

Functions of a Matrix - Untitled


Next: Specialized Solvers,
Previous: Matrix Factorizations,
Up: Linear Algebra


---

### 18.4 Functions of a Matrix

— Function File: **expm** (a)  
> Return the exponential of a matrix, defined as the infinite Taylor
> series
>
> ```
>           expm(a) = I + a + a^2/2! + a^3/3! + ...
> ```
>
> The Taylor series is *not* the way to compute the matrix
> exponential; see Moler and Van Loan, Nineteen Dubious Ways to
> Compute the Exponential of a Matrix, SIAM Review, 1978. This routine
> uses Ward's diagonal
> Pade'
> approximation method with three step preconditioning (SIAM Journal on
> Numerical Analysis, 1977). Diagonal
> Pade'
> approximations are rational polynomials of matrices
>
> ```
>                -1
>           D (a)   N (a)
> ```
>
> whose Taylor series matches the first
> `2q+1`
> terms of the Taylor series above; direct evaluation of the Taylor series
> (with the same preconditioning steps) may be desirable in lieu of the
> Pade'
> approximation when
> `Dq(a)`
> is ill-conditioned.

— Function File: **logm** (a)  
> Compute the matrix logarithm of the square matrix a. Note that
> this is currently implemented in terms of an eigenvalue expansion and
> needs to be improved to be more robust.

— Loadable Function: [result, error\_estimate] = **sqrtm** (a)  
> Compute the matrix square root of the square matrix a.
>
> Ref: Nicholas J. Higham. A new sqrtm for matlab. Numerical Analysis
> Report No. 336, Manchester Centre for Computational Mathematics,
> Manchester, England, January 1999.
>
>
>
> **See also:** expm, logm.

— Loadable Function: **kron** (a, b)  
> Form the kronecker product of two matrices, defined block by block as
>
> ```
>           x = [a(i, j) b]
> ```
>
> For example,
>
> ```
>           kron (1:4, ones (3, 1))
>                 =>  1  2  3  4
>                     1  2  3  4
>                     1  2  3  4
> ```

— Loadable Function: x = **syl** (a, b, c)  
> Solve the Sylvester equation
>
> ```
>           A X + X B + C = 0
> ```
>
> using standard lapack subroutines. For example,
>
> ```
>           syl ([1, 2; 3, 4], [5, 6; 7, 8], [9, 10; 11, 12])
>                => [ -0.50000, -0.66667; -0.66667, -0.50000 ]
> ```
